# Short Term Effect of Ivermectin on the Bacterial Microbiota from Fecal Samples in Chinchillas (*Chinchilla lanigera*)

**DOI:** 10.3390/vetsci10020169

**Published:** 2023-02-20

**Authors:** Xinyi Ma, Jing Li, Luo Yang, Haoqian Liu, Yiping Zhu, Honglin Ren, Feng Yu, Bo Liu

**Affiliations:** 1 Department of Clinical Veterinary Medicine, College of Veterinary Medicine, China Agricultural University, Beijing 100193, China; 2 China Agricultural University Veterinary Teaching Hospital (Beijing Zhongnongda Veterinary Hospital Co., Ltd.), Beijing 100193, China

**Keywords:** chinchilla, ivermectin, fecal microbiota, 16S rRNA

## Abstract

**Simple Summary:**

For the first time we evaluated the effect of ivermectin treatment on the fecal bacterial microbiota of healthy chinchillas by 16S rRNA gene sequencing. Ten clinically healthy chinchillas were included in this study. Via comparing the difference between microbiota composition of fecal samples of the 10 chinchillas before and 14 days after ivermectin administration, we found out that there was no significant alteration in the abundance and diversity of fecal bacterial microbiota after ivermectin injection, and the relative abundance of some bacterial species changed without observed negative impact on chinchillas’ health. These results indicated single subcutaneous injection of ivermectin in therapeutic dose to healthy chinchillas had a mild impact on the fecal bacterial microbiota in short term and provided guidance for clinical ivermectin treatment in chinchillas.

**Abstract:**

The gastrointestinal microbiota plays an important role in health of the host animals and the detrimental influence of pharmaceutical treatment on the fecal microbiota receives an increasing concern. The clinical use of ivermectin on chinchillas has not yet been evaluated. The purpose of our study was to assess the influence of ivermectin injection on the fecal bacterial microbiota of chinchillas. A with-in subject, before and after study was performed on 10 clinically healthy chinchillas during a 14-day period, all chinchillas received the same ivermectin treatment, and the microbiota from their fecal samples before and after administration were compared as two experimental groups. Fecal samples were collected on day 0 (before ivermectin administration) and day 14 (post ivermectin administration). Fecal bacterial microbiota was analyzed by bacterial 16S rRNA gene sequencing. No clinical abnormalities were observed post subcutaneous administration of ivermectin. No significant alteration was found in the abundance and diversity of fecal bacterial microbiota after treatment, but the dominant position of some bacterial species changed. In conclusion, ivermectin administration was associated with minimal alternations of the fecal bacterial microbiota in healthy chinchillas, and these changes had no observed negative effect on general health of chinchillas in short term.

## 1. Introduction

Chinchillas are non-ruminant herbivores that belong to Chinchillidae family, native to the Andes of South America. Although it is considered that wild chinchillas are almost extinct, domestic chinchillas descended from *Chinchilla lanigera* are often bred in fur farms because of their soft and fine furs or kept as companion exotic pets [1,2]. In chinchillas, *Syphacia obvelata* is occasionally seen, and ectoparasites such as *Ctenocephalides* spp., *Lagidiophthirus* spp., and *Atricholaelaps chinchillae* can be observed [3].

In recent years, more studies are focusing on the interaction between pharmaceuticals and gut microbiota, which can regulate the host metabolism and maintain as an important barrier for animals to prevent the colonization of pathogenic microorganisms [4,5]. Gut microbiota participates in the formation and breakdown of various compounds such as short chain fatty acids (SCFAs), organic acids, conjugated linoleic acid, phenolic compounds and so on, which play an important part in regulating metabolism, the immune system, and inflammatory response. Furthermore, via the regulation, gut microbial metabolites may affect the pathogenesis of a diverse range of diseases [5,6,7]. Thus, it is evident that changes in the gut microbiota have effects on the course of many diseases and affect host homeostasis and immune function [4,5,8].

Ivermectin is an antiparasitic, commonly used for controlling parasitic infestation in chinchillas by its oral or injectable formulation [3,9,10]. However, since it has antibacterial properties [11], the treatment of ivermectin may lead to the imbalance of gut microbiota, and that may promote the emergence and development of diseases [5,6,8]. A previous study found that combinative administration of fenbendazole and ivermectin tablets disturbed the homeostasis of gut microbiota and metabolism in Amur tiger [12]. One study on hookworm infected adolescents showed that combination treatment of tribendimidine and ivermectin triggered a re-composition of the gut microbiota [13].

At present, there is no report about the ivermectin effects on the fecal bacterial microbiota of chinchillas. Our study is the first to focus on the subcutaneous injection of ivermectin in chinchillas and explored its potential impacts on bacterial composition using 16S rRNA gene sequencing.

## 2. Materials and Methods

### 2.1. Animals

This study included 10 adult chinchillas (5 males and 5 females), ranging in age from 1–2 years, which belong to a commercial farm in Beijing, China. No animals participating in the study received any treatment prior to the experiment. Chinchillas were placed in individual cages, within a climate-controlled room temperature maintaining between 21 °C and 23 °C, and they were provided with a commercial pelleted diet (Mazuri^®^ Chinchilla Diets, Land O’Lakes, Inc., Melrose, MN, USA) with fresh timothy hay and tap water ad libitum. All animals were clinically healthy based on normal physical examination and close observation of food intake and fecal output. The shape and color of all fecal pellets during the experiment were normal, and for fecal samples collected from the 10 chinchillas on day 0 (before ivermectin injection) and day 14 (14 days after ivermectin injection), no gastrointestinal parasites were found in fecal fresh smear and zinc sulfate floating test under microscopic examination. Our study was approved by the animal care and use committee of China Agricultural University and we obtained the informed consent statement from the breeder.

### 2.2. Study Design and Fecal Sample Collection

Within subjects, we performed a before and after study instead of a cross over study given that the necessary washout period of ivermectin in chinchillas is unknown. In this experiment, the 10 chinchillas were injected subcutaneously with ivermectin injection (Ivomec^®^, Merial, Ingelheim am Rhein, Germany, 1.0% *w*/*v* sterile solution, dilute 10 times by saline) 0.4 mg/kg on day 0.

Fresh fecal samples were collected on day 0 and day 14, divided into day 0 group and day 14 group, respectively. Chinchillas were placed individually into clean cages, and fecal pellets were collected upon evacuation. Following collection, these samples were transferred immediately into sterile cryogenic vials by sterile forceps and stored at −80 °C until microbiota analysis [14].

### 2.3. DNA Extraction and 16S rRNA Sequencing

Total bacterial genomic DNA was extracted by using a commercially available extraction kit (PowerFecal^®^ DNA Isolation Kit, MO BIO Laboratories, Inc., Carlsbad, America) from fecal samples according to manufacturer’s instructions. To analyze the microbiota composition of bacterial communities in the fecal samples, the primer pairs from 338F (5′-ACTCTACGGAGCAGCA-3′) to 806R (5′-GACTACHVGGTWTCAT-3′) were used to amplify the hypervariable region V3 and V4 of bacterial 16S rRNA genes and the final products were sequenced by Illumina Novaseq6000 PE250 [15].

### 2.4. Statistical Analysis

To access the diversity of the gastrointestinal bacterial community (e.g., α-diversity), the number of observed operational taxonomic units (OTUs), the Chao 1 estimator and the ACE estimator assessed the richness of bacterial composition and represented the number of different taxa in both groups. By using USEARCH (v10.0), sequences with ≥97% similarity were assigned to the same OTUs and 0.005% of the number of sequences was used as the threshold to filter OTUs. In addition, the Shannon-wiener index and Simpson index were used for evaluating the abundance and evenness of samples, namely community diversity. The Chao 1 index, Ace index, Shannon index, and Simpson index were calculated by QIIME2 (v2021.4.0, Quantitative Insights Into Microbial Ecology 2).

To evaluate differences in overall bacterial microbiota composition between groups (i.e., β-diversity), it was measured by bray-curtis distance metric and visualized using Principal Coordinate Analysis (PCoA) plots to evaluate the similarity between two groups. Wilcoxon rank-sum test calculated the *p*-value of relative abundance difference to clarify whether the change was statistically significant, and the linear discriminant analysis effect size (LEfSe) figured out the significantly different biomarkers of the two groups.

A *p*-value of <0.05 was considered significant.

## 3. Results

### 3.1. Clinical Findings and Parasitic Examination

After administration of ivermectin, no chinchillas developed adverse reactions during the study. The appearance of feces was unchanged, being the same oval shape, dry and hard state. There was no loose stool or any clinical abnormalities. No parasites or eggs were found in fecal fresh smear and zinc sulfate floating test under microscopic examination before and after ivermectin treatment.

### 3.2. Microbiota Profile Analysis

**Figure 1:**
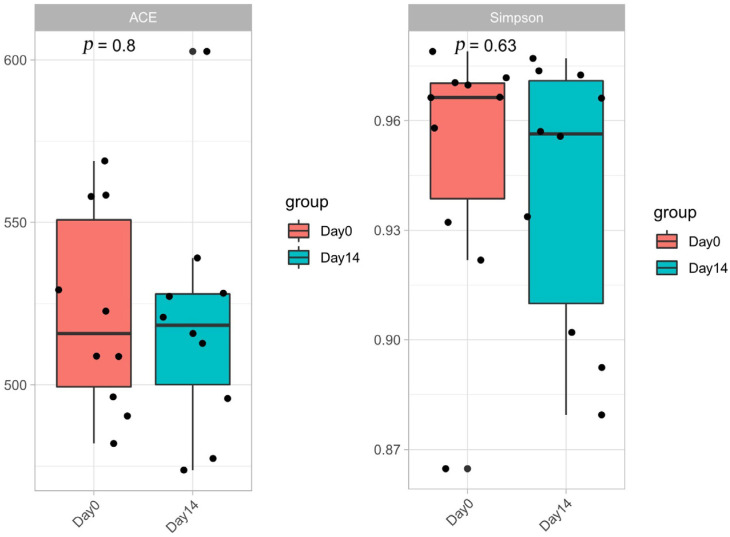
The ACE indexes (**left**) and Simpson indexes (**right**) of two groups, indicating the richness and diversity change after administration.

The number of OTUs in day 0 group and day 14 group were 684 and 662, respectively, and 646 identical OTUs were shared between the two groups. The richness and composition of intestinal microbiota were measured by α-diversity indexes, which contain the Chao 1 index, ACE estimator, Shannon index, and Simpson index. There was no significant difference of the microbiota abundance between day 0 and day 14 with the result of Chao 1 index (*p* = 0.843), ACE index (*p* = 0.823), Shannon index (*p* = 0.464), and Simpson index (*p* = 0.580) (Figure 1).

### 3.3. Compositional Analysis

**Figure 2:**
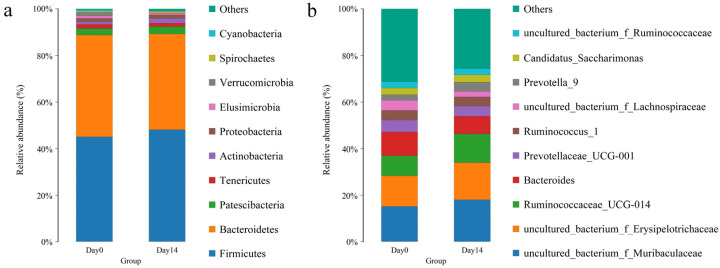
(**a**) The composition of fecal bacterial microbiota in day 0 and day 14 group at the phylum level. (**b**) The composition of fecal bacterial microbiota in day 0 and day 14 group at the genus level.

When comparing the taxonomic abundance of the two groups at the phylum level, the microbial community were similar before and after ivermectin administration. In both groups, Firmicutes was the most abundant phylum, followed by Bacteriodetes, Patescibacteria, Tenericutes, Actinobacteria, Proteobacteria, Elusimicrobia, Verrucomicrobia, Spirochaetes, and Cyanobacteria (Figure 2a). Firmicutes and Bacteriodetes were the main composition of bacterial phyla, which added up to 88.76–89.22%. Cyanobacteria was 0.29% on day 14, which decreased significantly compared to 0.73% on day 0 (*p* = 0.034).

For the genus level, 180 OTUs were identified, the most common bacterial genera were *uncultured_bacterium_f_Muribaculaceae* and *uncultured_bacterium_f_Erysipelotrichaceae* in both groups. The other common genera in the two groups were *Ruminococcaceae_UCG-014*, *Bacteroides*, *Prevotellaceae_UCG-001*, *Ruminococcus_1*, *uncultured_bacterium_f_Lachnospiraceae* and *Prevotella_9* (Figure 2b). Higher levels were found in *Bacteroides* (10.264%), *Prevotellaceae_UCG-001* (5.156%), *Ruminococcus_1* (4.177%), and *uncultured_bacterium_f_Lachnospiraceae* (4.435%) on day 0, with no significant difference compared to day 14.

β-diversity was accessed by a PCoA. On the basis of Bray–Curtis, no statistic difference in microbial community composition between day 0 and day 14 was founded at phylum level.

**Figure 3:**
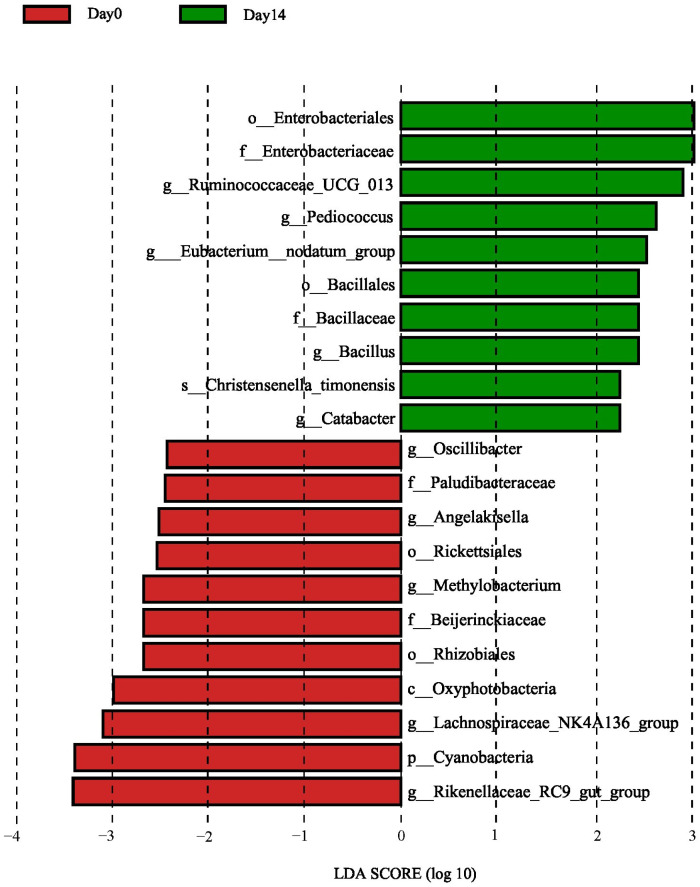
LEfSe showed the significantly different bacterial taxa of which the LDA scores were above 2 between day 0 and day 14 group.

The linear discriminant analysis (LDA) effect size was applied to compare the difference between two groups. At the genus or higher levels, there was significant difference (Figure 3) between the day 0 and day 14 groups (LDA score > 2, *p* < 0.05). The genus included in the specific biomarkers of the day 0 group was *Rikenellaceae_RC9_gut_group*, which also had the highest LDA scores. The rest of genera associated with the day 0 group were *Lachnospiraceae_NK4A136_group*, *Methylobacterium*, *Angelakisella*, and *Oscillibacter*. While *Ruminococcaceae_UCG_013*, *Pediococcus*, *Eubacterium*, *Bacillus,* and *Catabacter* were the genera most associated with the day 14 group.

## 4. Discussion

To our knowledge, this is the first study to assess the effect of ivermectin treatment on the fecal bacterial microbiota of chinchillas by 16S rRNA gene sequencing. Our pilot study only found minimal changes of fecal bacterial microbiota in chinchillas before and after ivermectin injection without significant difference in richness and diversity and no obvious clinical abnormalities were noticed.

The treatment of ivermectin did not change the main bacterial composition significantly at the phylum and genus level. According to previous research, Firmicutes, Bacteroidetes, Verrucomicrobia, Spirochaetes, and Proteobacteria were the main fecal microbiota in domestic herbivores, and the ratio of Firmicutes to Bacteroidetes was <2 in hindgut fermenters [16,17,18]. Our experimental result shared similarities that Firmicutes and Bacteroidetes dominated among these phyla and the ratio of them was <2 in chinchillas. Although the use of ivermectin did not alter the main phyla composition of fecal bacterial microbiota, the relative quantity and proportion of bacterial microorganisms in the two groups were still different in some ways.

At the phylum level, the significant change was decreased Cyanobacteria post ivermectin administration. Cyanobacteria exists widely in surrounding environment like marine and freshwater and has also been detected in the fecal bacteria communities of horses and volcano rabbit [19,20,21], which some similarity in hindgut fermenters. As a minor component of intestinal microflora, one study hypothesized that the Cyanobacteria overgrowth with production of neurotoxins had been related to the development of neurodegenerative diseases such as Equine Motor Neuron Disease [22]. Additionally, there is an overview showing that many diseases such as virus infection, neurodegeneration, gastrointestinal and metabolic diseases are associated with higher gut Cyanobacterial abundance [19]. Although Cyanobacteria are always associated with hazardous production and negative influence, there are potentially beneficial compounds produced by Cyanobacteria [23]. Besides, some studies also showed that cirrhosis and obesity were related to lower gut Cyanobacterial abundance [19]. According to these previous studies, further research is needed to clarify the correlation between Cyanobacterial abundance and animal health. Our study for the first time found Cyanobacteria was a main component in chinchilla fecal microbiota, and ivermectin administration was related to its decrease. Considering the double-edged effects of Cyanobacteria, its potential effects on chinchillas’ intestinal health deserve further study.

For the genus level, *Uncultured_bacterium_f_Muribaculaceae* was the most common genus, found in the rumen microbial community of sheep as well [24], being slightly more predominant on day 14 post ivermectin usage in this study. This bacterial genus could produce succinic acid as an important gluconeogenic intermediate and was helpful with polysaccharides degradation and metabolism [25]. *Prevotellaceae_UCG-001*, however, was more predominant on day 0. As a negative fermentation parameter [26], when combined with higher *uncultured_bacterium_f_Muribaculaceae* in day 14, suggesting it is not a negative change for production and fermentation [24]. *Bacteroides* participates in the utilization of plant polysaccharide in marine and terrestrial herbivores [16]. It was slightly decreased on day 14 after ivermectin injection, meaning ivermectin might pose a negative influence on the plant utilization.

LEfSe analysis showed that the relative abundance of some bacteria changed statistically after administration. *Rikenellaceae_RC9_gut_group* is included in Rikenellaceae family, which produces hydrogen and mediates suppression of proinflammatory cytokines, was found to be associated with day 0 [27]. Besides, this genus participates in degrading plant-derived polysaccharides, and its relative abundance is positively associated with dietary fiber content [28]. *Oscillibacter* is a common genus in herbivores and may assist in plant polysaccharide utilization [16], was identified on day 0. Some articles showed that *Oscillibacter* contained beneficial bacteria, of which the metabolites like butyric acid and alpha-linolenic acid can resist inflammation and protect intestinal mucosa [29,30]. Speculating effects of ivermectin on these genera may affect the intestinal microbiota balance and change the fiber metabolism, which reduces the protective function of normal microbiota.

*Ruminococcaceae_UCG_013* is a genus in the Ruminococcaceae family, belongs to Firmicutes, and is most significantly associated with the day 14 group. This genus produces butyrate, promoting the differentiation of Treg cells which participate in enhancing the barrier integrity of epithelium and anti-inflammatory action, thus *Ruminococcaceae_UCG_013* is beneficial to intestinal immune function and general health [31]. In addition, *Ruminococcaceae_UCG_013* can help utilize or degrade indigestible fiber, such as cellulose and hemicellulose, and is an important genus for herbivores digestion [31,32]. Besides, *Pediococcus*, *Eubacterium,* and *Bacillus* were also related to day 14 significantly. *Pediococcus* is a genus of lactic acid bacteria, which are common intestinal microorganisms. This genus can be used as probiotics as *Pediococcus* strains can produce pediocin, an effective antilisterial bacteriocin. It has been proven that some *Pediococcus* strains isolated from horse feces have great inhibition on *Escherichia coli*, *Salmonella enteritidis*, *Listeria monocytogenes,* and *Staphylococcus aureus* [33]. *Eubacterium* is a common butyrate-producing genus in herbivore intestine [16], positively related to the level of SCFAs, which are beneficial to intestinal health of animals and play a key role in colon motility, immune regulation and intestinal inflammation inhibition [34,35]. *Bacillus* belongs to Firmicutes, plays a significant role in animal immunity and antibacterial ability. It is often used as probiotics and has various related commercially available products [36,37,38]. A *Bacillus* strain isolated from donkey cecum showed good ability on inhibiting the development of pathogens [38].

In chinchillas, gastrointestinal disorders are common and are associated with dysbacteriosis [39]. *Syphacia obvelata* is an internal parasite occasionally observed in chinchillas and ivermectin is a clinical treatment for the disease. However, ivermectin has antibacterial properties, which may lead to gastrointestinal disease by causing dysbacteriosis [8,11], so figuring out the influence of ivermectin on the gut microbiota of chinchillas is important.

Our study suggested that single subcutaneous injection of ivermectin in therapeutic dose had mild impact on the fecal microbiota of healthy chinchillas, and the result can provide guidance for clinical roundworms treatment in chinchillas. Considering one of the limitations of this study is the before-after study design, additional studies employing a non-treated control, or an interrupted time series analysis would be needed to validate our findings.

## 5. Conclusions

Overall, single subcutaneous injection of ivermectin had a mild impact on the fecal microbiota in healthy chinchilla and no significant passive influence was observed on general health in short term. Of the phylum, Cyanobacteria decreased significantly after treatment. Of the genera, *Rikenellaceae_RC9_gut_group*, *Lachnospiraceae_NK4A136_group*, *Methylobacterium*, *Angelakisella,* and *Oscillibacter* were associated with day 0, while *Ruminococcaceae_UCG_013*, *Pediococcus*, *Eubacterium*, *Bacillus,* and *Catabacter* were associated with day 14. Further investigations are warranted to clarify the influence of ivermectin on chinchillas with parasites infection or in a long term.

## Data Availability

The data presented in this study are openly available in Figshare at https://doi.org/10.6084/m9.figshare.21972641.v1 (accessed on 29 January 2023).
